# Biopolymer-assisted synthesis of Ag–Cd nanoparticles on chitosan matrix for catalytic removal of cationic dyes from water

**DOI:** 10.1038/s41598-026-49289-2

**Published:** 2026-05-07

**Authors:** Umer Younas, Sehrish Arif, Faisal Ali, Arooj Asif, Aimon Saleem, Faiza Hassan, Muhammad Pervaiz, Gaber E. Eldesoky, Giuseppe Mele, Awais Ahmad

**Affiliations:** 1 Department of Chemistry, The University of Lahore, Lahore, Pakistan; 2 Department of Engineering for Innovation, University of Salento, via per Monteroni, Lecce, 73100 Italy; 3 Department of Chemistry, Government College University, Lahore, Pakistan; 4 Department of Chemistry, College of Science, King Saud University, P. O. Box 2455, Riyadh, 11451 Saudi Arabia; 5 Faculty of Engineering and Technology, Shinawatra University, Sam Khok, Thailand

**Keywords:** Bimetallic nanoparticles, Catalysis, Chitosan, Kinetics, Biopolymer, Biochemistry, Biotechnology, Chemistry, Environmental sciences, Materials science, Nanoscience and technology

## Abstract

Metal nanoparticles are being used for different applications especially for the removal of toxic pollutants from different environmental segments. In this study, chitosan supported AgCdO nanoparticles have been prepared for the removal of toxic dyes from aqueous medium. Chitosan supported AgCdO nanoparticles (AgCdO-BMNPs@Ch) were synthesized and characterized using different techniques including UV/Vis, FT-IR, XRD and SEM to assess their formation as well as morphology. Crystallite size of bimetallic nanoparticles and composite was found to be 42.15 nm and 38.23 nm respectively. Particle size of nanoparticles as well as composite was also calculated using SEM images and flower Dahlia-like morphology of AgCdO nanoparticles was observed while the composite displayed colonies. In addition, the samples were than studied for catalytic degradation of cationic dyes Malachite green, Rhodamine-B and Methylene blue (MG, Rh-B and MB). Removal of the dyes via catalytic degradation using synthesized samples was recorded up to 91.62 %, 92.42 %, 96.16 % for RhB, MG and MB respectively. Performance of samples was also tested under different conditions and their recyclability was also evaluated. On the basis of current study, authors recommend the preparation of biopolymer supported bimetallic nanoparticles to improve their stability as well as efficiency.

## Introduction

Nanotechnology has been recognized as innovatory field that has revolutionized different areas of research in the last decade. The applications of nanomaterials in medicines, energy storage devices, agriculture, biotechnology, optical devices and catalysis etc., have contributed towards improving human life^1^. Based on the morphology and other characteristics, there are different nanoparticles (NPs), such as carbonaceous, metallic and polymeric etc.^2^, that are being designed and synthesized keeping in view the their applications. Metallic NPs have been used in different applications due to distinctive surface characteristics, size and shapes^2^, which make them effective in a number of fields especially catalysis^3^. Synthesis and catalytic potential of monometallic NPs have been reported by different researchers. Later on, bimetallic nanoparticles (BMNPs) were recognized as remarkable materials due to additional properties that are exhibited exclusively due to synergistic association of two different metals and improved catalytic potential is observed as compared to monometallic nanoparticles. Interaction between the two metals contribute towards improving their properties synergistically that ultimately enhance their performance in different reactions involving catalysis^4^. In recent studies, different bimetallic NPs have been reported for catalytic applications to remove different pollutants from aqueous medium^5,6^.

Among different metal NPs, silver NPs have been synthesized and investigated for numerous applications. Silver NPs have drawn the interest of experts due to different morphological features that are suitable for biological as well as catalytic activities^7^. Silver NPs have been successfully tested against toxic pollutants (heavy metals and organic materials) found in wastewater. The catalytic role of Ag-NPs for environmental remediation has been widely recognized by different researchers. Removal of toxic pollutants from aqueous medium, *i.e.* degradation (oxidation) of pesticides and dyes, using Ag-NPs has been studied and recommended in many studies^8,9^. In the same way, cadmium (Cd) as semiconductor possesses unique optical and optoelectrical capabilities that can be utilized to conduct photocatalytic processes^10^. Absorption and emission of visible light radiations are specific photosensitive properties that are necessary for any material for photocatalytic, photovoltaic, and biomedical applications. Considering different reports, Cd-NPs can be considered suitable catalyst for the removal of toxic pollutants i.e. dyes^11,12^. Recently, green synthesis of Ag and Cadmium based nanocomposite has been reported for the degradation of brilliant green dye and its efficiency was claimed higher as compared to Ag-NPs^13^. Bimetallic NPs of Ag-Au and Fe-CdO has been synthesized followed by evaluating their catalytic potential against different dyes^14,15^.

Cadmium-based materials are recognized in possessing potential health and environmental risks due to the likely release of Cd²⁺ ions. Cadmium is a highly toxic heavy metal which can accumulate in biological systems and may cause severe damage to liver, bones and kidneys upon prolonged exposure. The potential leaching of Cd²⁺ from Cd-containing nanomaterials during catalytic processes must be cautiously considered. Therefore, several approaches are frequently utilized, including the immobilization of Cd-based NPs on supporting matrices and the development of composite structures. Also, functionalization or surface coatings can substantially reduce Cd²⁺ ion release by stabilizing the NPs surface and limiting direct contact with the reaction medium. For example, polymer coatings or surface carbon have demonstrated to efficiently suppress the leaching of Cd²⁺ from CdO NPs and thus reduce its environmental toxicity^16,17^.

CdO is an n-type semiconductor with a bandgap of ~ 2.2–2.5 eV with higher electrical conductivity therefore it is suitable for the environmental remediations such as catalytic and photocatalytic applications^18^. Although, pure CdO often suffers from recombination of photogenerated electron-hole (e^−^/h^+^) pairs which suppresses its catalytic activity therefore, by combing it with different metals such as Ag not only enhances the charge separation but also form metal-semiconductor junction at the interface. Combining Ag and CdO can provide (i) higher surface reactivity, (ii) reduce e^−^/h^+^ recombination, (iii) improve light absorption and (iv) enhance charge separation^19^.

Chitosan is a non-toxic, biologically compatible, and ecological biopolymer that is derived from chitin and it is used in diverse fields such as food, medication, antimicrobial films, and coatings (such as smearing layers on fruits or vegetables) due to its antibacterial and antioxidant properties^20^. Chitosan has been studied as a support material due to presence of specific functional groups, biodegradability, and ability to form stable composites with metal NPs. Chitosan has been used for the preparation of different metal composites^21^ that served two major purposes. These biopolymer based composites possess potential to adsorb organic pollutants and it can also act as catalyst for the degradation of various toxic dyes^22^. There have been studies on the catalytic reduction of dyes using metal NPs supported on different biopolymers^23^.

In current study chitosan supported bimetallic AgCdO-NPs have been synthesized to form AgCdO-BMNPs@Ch nanocomposite. Recent literature has shown that CdO and Chitosan based nanorods were prepared for supercapacitance applications^24^. Similarly, CdO-TiO_2_@Ch was synthesized for photodynamic and photocatalytic studies^25^. In another study, Ag-CdO thin films have been prepared for the structural and optical properties^26^. Subramanian et al., studied Ac/Ag/CdO nanomaterials for the photocatalytic degradation of alizarin red dye^27^. No study has discussed the prospects of synthesizing a nanocomposite of Ag/CdO-BMNPS@Ch. Therefore, it is safe to say that this novel composite has never been reported prior, up to the best of our knowledge, and the catalytic studies have never been reported for the said composite. The as-synthesized sample of AgCdO-BMNPs@Ch was characterized using FT-IR, UV-Vis, SEM and XRD analysis. Chitosan supported AgCdO NPs were tested as catalyst for the degradation of cationic dyes (malachite green (MG), rhodamine-B (RhB) and methylene blue (MB)). In addition, effect of different parameters including time, pH and temperature on dye removal potential of Ch-AgCd NPs was also studied.

## Materials and methods

### Materials

All of the chemicals used in the studies were of analytical grade. The chemicals including AgNO_3_ (99 % purity), NaBH_4_ (99 % purity), CdCl_2_ (99 % purity), MB dye (97.5 % purity), RhB dye (97.5 % purity), MG dye (97 % purity) and chitosan (> 97 % purity) were purchased from Sigma-Aldrich, UK. All of the reagents and metal precursors were employed exactly as they were in their original conditions, with no additional processing.

### Synthesis of AgNPs, CdONPs and AgCdO-BMNPs

Silver NPs were synthesized by adding 5 mL tri-sodium citrate (0.05 M) and 5 mL silver nitrate (0.05 M) into 180 mL cold distilled water (5–10 °C). The solution swirled for 15 min. After that, 5 mL of sodium borohydride (0.05 M) gradually poured down. With sodium hydroxide (1 M), the pH was set to 11. The Ag NPs colloids were stored in glass vials^28^.

Cadmium oxide nanoparticles (CdO NPs) were prepared by adding 50 mL (0.05 M) cadmium chloride in 100 mL distilled water. With steady stirring, the NaOH (1 M) solution was added dropwise to the solution until a pH 11 was obtained. The snowy precipitates that had developed were settled overnight. After that, it was filtered and rinsed 3–4 times with distilled water and ethanol. Nanoparticles formed were dried at 100 °C and calcined for 2 h^29^.

In a beaker, 50 mL each of 0.01 M CdCl_2_ and 0.05 M AgNO_3_ were heated on a hot plate for half an hour with continual stirring. Afterwards, trisodium citrate (0.05 M) was added dropwise. The color change from colorless to dark brown confirmed he formation of said Ag/CdO particles The resulting AgCdO nanoparticles were separated by centrifugation and dried in an oven at 100 °C^30^.

### Synthesis of AgCdO-BMNPs@Ch

Firstly, 1% chitosan solution was prepared by adding 0.5 g of chitosan in 50 mL distilled water, then few drops of glacial acetic acid was added to completely dissolve the natural polymer and agitated for 2 h^31^. Afterwards, 50 mL solution of pre-synthesized AgCdO-BMNPs was added to the above solution and stirred for an hour at 60 °C. Then, 5 mL sodium borohydride (0.01 M) was added to the above reaction mixture followed by the addition of sodium hydroxide (1 M) to maintain the pH between 10 and 11, with continuous stirring for an hour at 200 rpm. A thick gel like consistency was formed which then centrifuged for 30 min followed by washing with a mixture of ethanol and distilled water. The obtained product was oven dried at 60 °C for 24 h and kept in a glass vial for future use.

### Characterization of the synthesized BMNPs and the composite

Characterization of the synthesized samples was performed using techniques such as UV-Vis, FTIR, SEM and X-ray diffraction. UV-Vis absorption spectra were recorded in the wavelength range of 200–800 nm using a double beam spectrophotometer (Shimadzu, UV-1800, Japan) to characterize the fabricated bimetallic nanoparticles and the composite. FTIR spectrum (IR Prestige 21, Shimadzu, Kyoto, Japan) was used to explore the existence of various functional groups. Analytical X’ Pert PRO X-ray diffractometer was used to obtain X-ray powder diffraction patterns of synthesized samples. Structure morphology of was determined using SEM technique, and regions varying in width from 1 cm to 5 μm were scanned in a scanning mode (magnification ranging from 20X to approximately 30,000X, spatial resolution of 50 to 100 nm).

### Catalytic degradation of cationic dyes

The time-dependent catalytic degradation process was monitored using UV-Vis spectrophotometer. The reactions were observed to assess the best optimized conditions for the reduction of cationic dyes (MG, RhB and MB). The standard calibration curve using different concentrations of dyes ranged from 0.1 to 0.9 mM was made to find the best optimized concentration of dyes. Then, the 1 mM NaBH_4_ solution was used against RhB and MG dyes, and 2 mM NaBH_4_ for MB dye was used and in each solution 0.05 mg/mL nanocatalyst was. Furthermore, the 0.1 M HCl and 0.1 NaOH have been used to maintain pH conditions to assess the optimum catalytic degradation environment.

## Results and discussion

### Spectral analysis and functional group detection

**Fig. 1:**
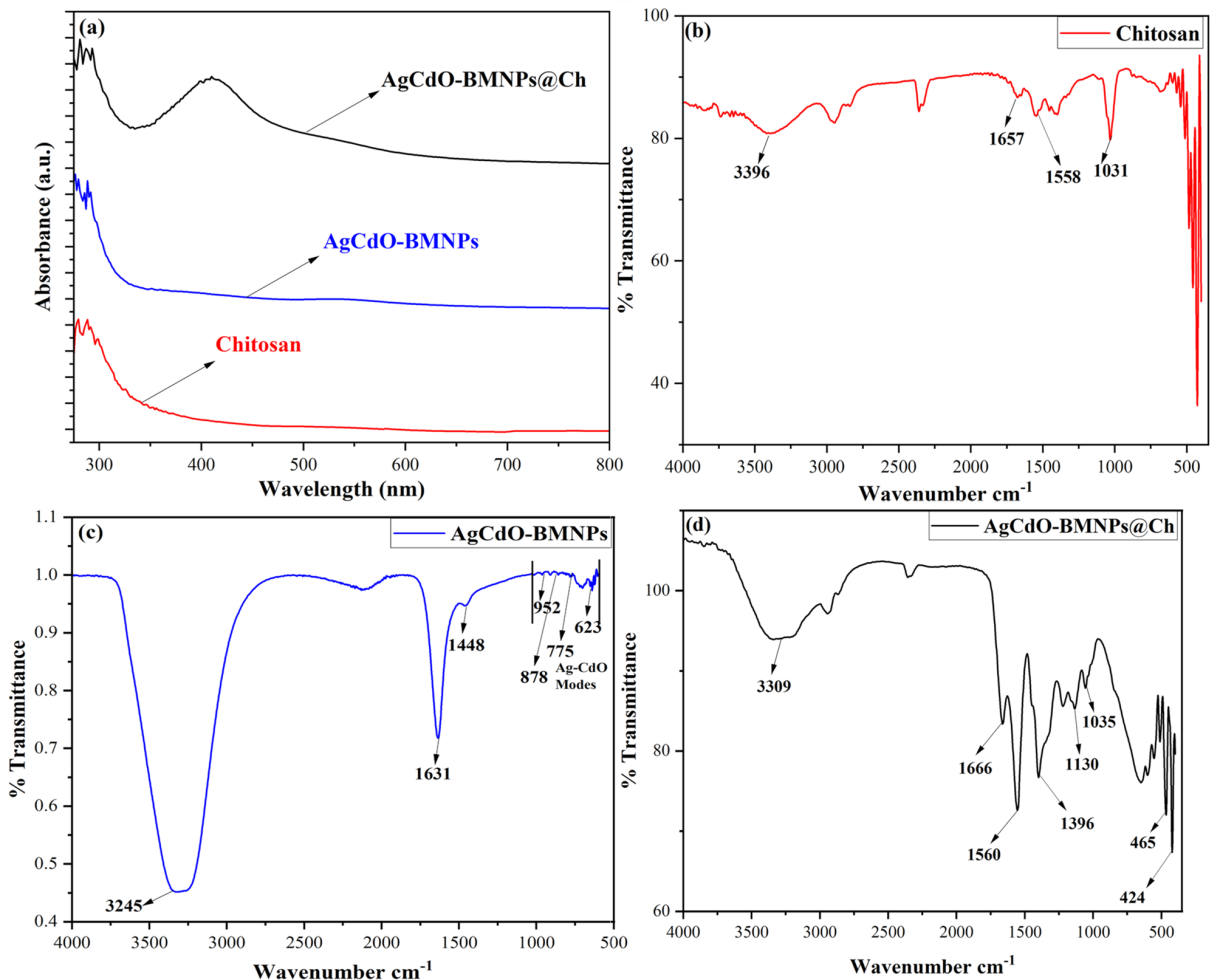
(**a**) UV/Visible of Ch, AgCdO-BMNPs, and AgCdO-BMNPs@Ch; (**b**–**d**) FTIR of Ch, AgCdO-BMNPs, and AgCdO-BMNPs@Ch respectively.

The optical absorbance of Chitosan (Ch), BMNPs and the composite was recorded (Fig. 1a). The Ch exhibits unique properties due to the presence of 2–amino–2–deoxyglucose units which are the backbone of Ch. Its peaks can be observed in between ranges 250–430 nm. The peak of Ch appears at 283 nm with some minor peaks at 277 and 288 nm^32^. Similarly, peaks observed in between 270 and 285 nm could be red-shifted peak of Ag-CdO appeared due to the transition of n–π* electrons. It could also be due to the increase in surface scattering and grain boundary of the BMNPs^33,34^.

The FTIR analysis of Ch, AgCdO-BMNPs, and AgCdO-BMNPs@Ch are given in Fig. 1(b-d). The spectra are recorded from 4000 to 400 cm^− 1^. The spectrum in Fig. 1d corresponds to the band at 3396 cm^− 1^ is attributed to the stretching vibrations of –NH_2_ and O–H group. The bands at 1657 and 1558 cm^− 1^ are ascribed to CONH_2_ and NH_2_ groups correspondingly, similar results were reported by^35^.

Similarly, Fig. 1c explained various functional group of BMNPs of Ag and CdO. The peak at 3245 cm^− 1^ corresponds to the stretching vibrations of Cd(OH)_2_. Bands at 1631 cm^− 1^ and 1448 cm^− 1^ are attributed to the bending and asymmetric modes of water molecules respectively. The band at 878 cm^− 1^ corresponds to the asymmetric carboxyl ions stretching modes. The absorption bands at 775 and 623 cm^− 1^ designated to the coordination of Ag with CdO and the formation of Cd–O bond, similar results were reported by^36–38^. The composite’s spectrum is shown in Fig. 1d, bands at 3309 and 2935 cm^− 1^ are ascribed to OH and NH stretching vibrations where the latter one is the new shoulder peak ofamide B, which confirms the interactions between Ch and AgCdO BMNPs. While the shift in dips at 1666 from 1657, 1560 from 1558 and the emergence of new peak at 1396 cm^− 1^ is attributed to the stretching vibrations of C = O, bending vibrations of NH and wagging modes of CH_2_ respectively confirms the chelation of BMNPs with the OH and amine groups of Ch^39,40^. Hajji and his coworkers reported the interactions of Ag, Cd, O and N results in the shift in the peaks and the decrease in the intensities of the bands^41^. The peaks at 1130 and 1035 cm^− 1^ are designated to the C–O–C anti-symmetrical linkages of the chitosan moieties whereas the peaks in between 1000 and 450 cm^− 1^ confirmed the out-of-the-plane OH and CH groups deformation of the composite. All the other peaks in the said region confirmed the formation of coordinate bonds between Ch, hydroxyl, amino, Ag and Cd groups, similar results were reported by^42,43^. In the FTIR spectrum of the AgCdO–chitosan nanocomposite, the band observed around ~ 400 cm⁻¹ can attributed to the metal–oxygen lattice vibrations (Ag–O/Cd–O) of the AgCdO phase, confirming the successful formation and incorporation of the metal oxide within the chitosan matrix. This low‑wavenumber feature, together with the characteristic chitosan bands (e.g., O–H/N–H stretching and C–O–C vibrations), indicates effective interaction between AgCdO nanoparticles and the polymer chains, which can contribute to the improved structural stability and functional properties of the composite.

### Structural and morphological aspects

**Fig. 2:**
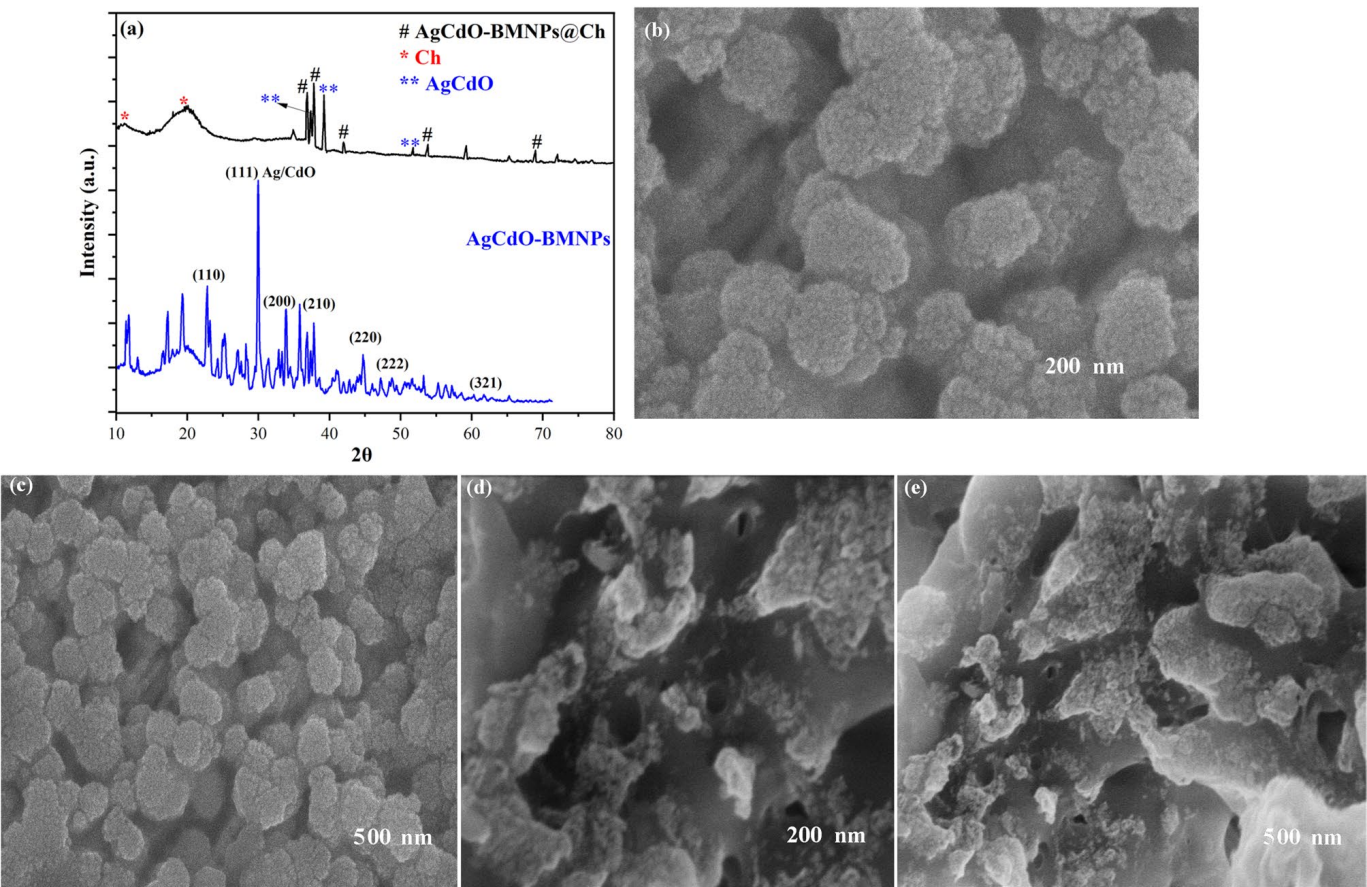
(**a**) X-ray diffractogram of BMNPs and composite, (**b**,**c**) SEM micrographs of BMNPs and (**d**,**e**) SEM images of AgCdO-BMNPs@Ch.

The structural characteristics of BMNPs and the composite are given in Fig. 2a. The X-ray diffractogram displayed sharp and intense peaks which match well with the PDF reference # 01-078-0134. The miller planes reported at (110), (111), (200), (210), (220), (222) and (321) confirmed the cubic crystalline geometry of the pristine AgCdO BMNPs. The average grain size was calculated using Debye-scherrer equation: D = Kλ/βcosθ, where D is the average crystallite thickness, K is the Scherrer constant (0.94), λ is the X-ray irradiation range (0.15406 nm), β is the FWHM in radians, and θ is the diffraction angle in radians. The mean crystallite size of calculated was 42.15 nm using the quantitative value of each item. The intensity peaks confirmed to be the highest degree of crystallinity in the said sample. The diffractogram revealed the predominantly grown (111) plane in comparison to other diffracted planes confirming the crystallized orientation of AgCdO BMNPs. The interaction between chemisorbed oxygen ions on the surface of AgCdO led to the liberation of electrons to the conduction band which results in change in the peak intensities, similar results were observed by^44,45^. Note that only high intensity peaks were used to calculate the grain size of BMNPs.

The XRD analysis was employed to elucidate the crystal structure of the composite shown in Fig. 2a. The characteristic peaks of Ch were observed at 10.1° (020) and 19.5^ο^ (110) while other significant peaks were observed at 36.81° (100), 37.73° (002), 39.21° (101), 41.15° (102), 51.71° (103), 53.63° (200), 65.21°(112), and 72.81° (201) confirmed the hexagonal crystal structure of the as-synthesized composite with a JCPDS reference # 01-065-0184. The characteristic peaks at 36.81° and 37.73° confirmed the crystalline nature of polymeric BMNPs. The crystallite size of the composite calculated using the Debye–Scherer formula explained earlier comes out to be 38.23 nm (for calculation, only sharp crystalline peaks were considered), similar results were reported in previous studies^46,47^. While peaks at 37.27°, 39.25° and 51.52° coincides with the peaks present in Ag/CdO confirming the correlation between the BMNPs and the composite itself.

The morphological aspects of BMNPs and composite are given in Fig. 2b–e. The SEM micrographs of BMNPs showed flower Dahlia-like morphology (Fig. 2b and c) which can be interpreted as quasi-spherical to irregular granular with noticeable agglomeration, while the composite displayed porous, sponge-like and irregular network structure as can be seen Fig. 2d and e. These types of aggregates are commonly observed in the metal-oxide NPs because they have strong interparticle interactions and high surface energy ^48,49^. After the incorporation of BMNPs, a visible transformation in morphology has been observed. The BMNPs were uniformly distributed and partially embedded within the Ch-framework confirming their interaction with the polymeric matrix, therefore stabilizing the composite ^50^. The particle size of AgCdO and AgCdO-BMNPs@Ch was calculated using image j software. For the composite, both smaller and larger particles were separately calculated. The particle size of AgCdO calculated is 75 nm while that of AgCdO-BMNPs@Ch is 52 nm for smaller particle and 12 μm for larger particles, similar results were reported by^51^.

The crystallite size calculated for BMNPs was 42.15 nm while that of AgCdO-BMNPs@Ch is 38.23 nm while the particle size for the former one is 75 nm and the later one has two different sizes (i) primary particles (52 nm) and (ii) secondary particles  (large agglomerates) (12 μm) respectively. The difference between these values confirmed that the particle is composed of multiple crystallites aggregated together forming a polycrystalline structure; various literature validated the variation in the size obtained from XRD to SEM^52^. Whereas, the composite has two types of particles as mentioned earlier, confirmed the behavior of Ch-matrix as stabilizing and capping agent which limits the growth of crystal during nucleation^53,54^. The large agglomerates (12 μm) does not show any individual NPs but they are the secondary agglomerates formed by the embedment of multiple BMNPs into the Ch-matrix which could be due to the polymer chain entanglement or due to interparticle interactions, as confirmed by^55^.

### Catalytic studies

#### Evaluating the degradation potential of Nanocatalyst

**Fig. 3:**
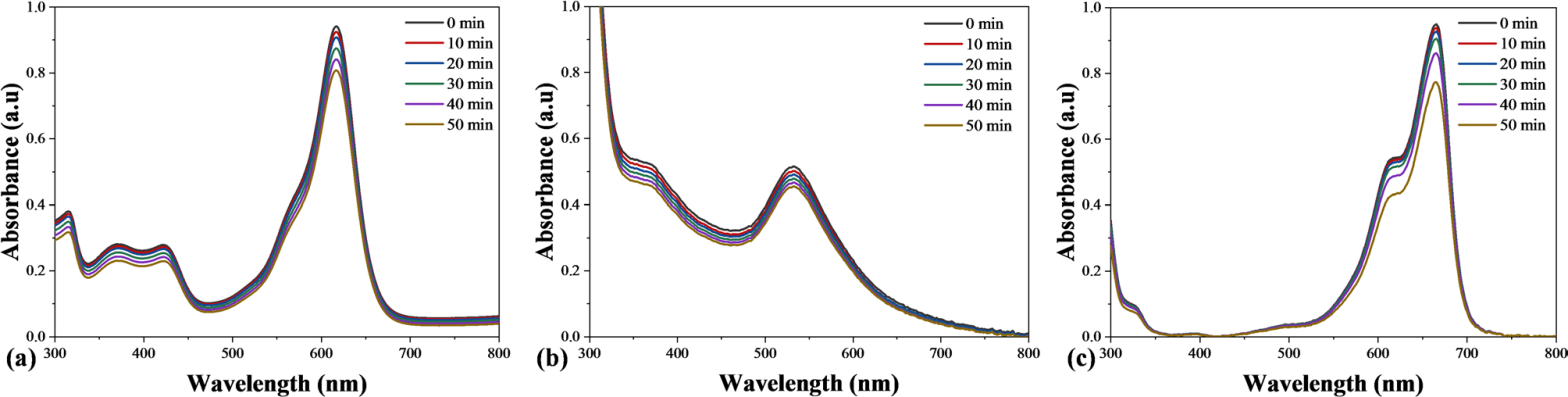
UV-Vis spectra of degradation of (**a**) MG, (**b**) RhB and (**c**) MB dyes without the nanocatalyst but with NaBH_4_ at optimized conditions.

The degradation potential of AgCdO-BMNPs@Ch was assessed in the absence of reducing agent (NaBH_4_) (Fig. 3). At first, the reaction was performed without the addition of nanocatalyst but in the presence of NaBH_4_ and i dyestuff solution it occurred and to ensure the reduction of dyes, conditions were optimized.Initially no reduction was observed in all three of the dyeseven after about 1 h of the reaction.The reduction of MG, MB, and RhB was still not viewed even after an hour in the absence of AgCdO-BMNPs@Ch but a slight decrease in the λ_max_ of the dyes solution alone was experimented. The spectra of all three dyes are given in the Fig. 3a–c. The degradation of dyes was only observed when the mixture of dyes and reducing agent were aided with the nanocatalyst. It could be due to the increase in the rate of reaction and decrease in the energy of activation. Catalysis occurred at the surface of the BMNPs whereas the increased surface area also enhances the catalytic efficiency^56^.

#### Catalytic degradation of cationic dyes using AgCdO-BMNPs@Ch

**Fig. 4:**
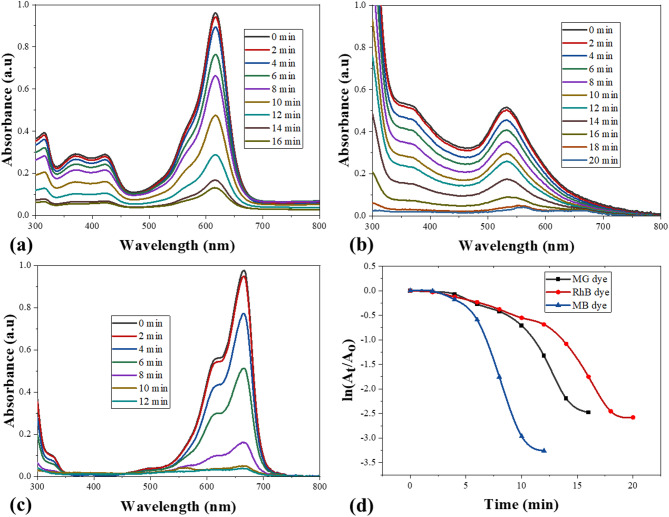
UV-Vis spectra of catalytic degradation of (**a**) MG, (**b**) RhB and (**c**) MB dyes using AgCdO-BMNPs@Ch and NaBH_4_ at optimized conditions whereas (**d**) Kinetic studies of the said dyes.

The degradation potential of AgCdO-BMNPs@Ch was investigated using different cationic dyes (MB, MG and RhB) at optimum conditions followed by the usage of a reducing agent NaBH_4_. For MB dye, the nanocatalyst taken was 0.07 mL (0.05 mg/mL), while the concentration of dye used was 1.5 mL (0.02 mM) followed by 0.5 mL (2 mM) reducing agent respectively. Similarly, 1.5 mL (0.01 mM) MG dye, 0.07 mL (0.05 mg/mL) of the nanocatalyst and 0.5 mL (1 mM) of NaBH_4_ were used. For RhB dye, 1.5 mL (0.025 mM) of dye, 0.07 mL (0.05 mg/mL) of the catalyst and 0.5 mL (1 mM) of NaBH_4_ were used. The degradation of the MG, MB and RhB by nanocatalyst in the presence of NaBH_4_ was confirmed by the continuing decrease in the intensity of the absorption peaks of MG, MB and RhB. The green, blue and pink colors were completely discharged within 16, 12 and 20 min respectively. The reaction time is indicated in Fig. 4a–c. The completion of reactions is observed with asolution which could be due to the π–π* transitions of azo groups or xanthene conjugates which were no longer present whereas the reaction without NaBH_4_ did not show a considerable change in the intensities as confirmed via Fig. 3 with an observation period of 50 min for all three of the dyes. The degradation could be the result of the breakage of –N = N– into –NH_2_ group in MG and MB where a cleavage of N-dethylation of the RhB to convert the dyes into less toxic forms. These results are in good accordance with the studies of Pal et al. regarding the utilization of nanocatalyst of AgNPs for the reduction of aromatic nitro into analogous amino compounds in the presence of NaBH_4_^57^.

The order of catalytic degradation of cationic dyes in terms of time completion was MB > MG> RhB. The dyes were degraded catalytically and then their % degradation and kinetics were evaluated using absorption values. All three dyes followed pseudo 1^st^ order kinetics with Langmuir-Hinshelwood mechanism of catalysis as shown in Fig. 4d. The reason for this order of reactivity of the catalytic reactions was the usage of NaBH_4_ (10 times more) as compared to the substrate (dye) by following the expression ln(A_t_/A_o_)=-k×t. In this condition, the order of reaction depends primarily on the concentration of the reactant (dye) rather than the reductant (NaBH_4_)^58^. Secondly, the higher amount of NaBH_4_ speeds up the reaction, which makes the reaction time efficient. The order for fastest to slowest catalytic degradation pattern was MB > MG> RhB with rate-constant (k) values of 0.4762 min^− 1^, 0.2967 min^− 1^ and 0.2054 min^− 1^ respectively (Fig. 4d). This quantitative analysis of the degradation of the dyes will help us understanding the speed of reaction in terms of time dependency, rate constant (k) and to evaluate the catalytic potential of AgCdO-BMNPs@Ch in the presence of NaBH_4_.

#### Percentage degradation and recyclability

**Fig. 5:**
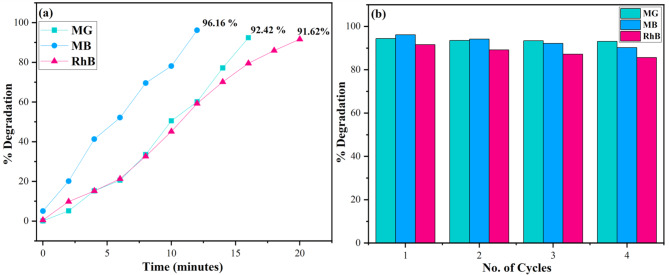
(**a**) % degradation of dyes with NaBH_4_, (**b**) Stability of the catalyst under optimized conditions.

The % degradation was calculated as shown in Fig. 5a. The degradation of RhB was 91.62 % while for MG it is 92.42 % and for MB dyestuff it is the highest 96.16 % as shown in Fig. 5a. The catalytic efficiently of the nanocatalyst was calculated for multiple cycle and were documented in Fig. 5b. After 4 cycles, the catalyst along with the reducing agent does not substantially lose its degradation efficiency, giving 94.42, 93.51, 93.42 and 93.1 % in the 1^st^ to 4^th^ cycles for MG dye. While for MB, the recyclability turned out to be 96.16, 94.12, 92.15 and 90.21 % for 4 consecutive cycles. For RhB, the values calculated were 91.62, 89.15, 87.21 and 85.65 % for 1^st^, 2^nd^, 3^rd^ and 4^th^ cycles, see Fig. 5b. Based on these results, we can say that the catalyst is most stable for the catalytic degradation of various dyes and it also has the potential to degrade other organic pollutants^59,60^.

#### pH and temperature factor studies

**Fig. 6:**
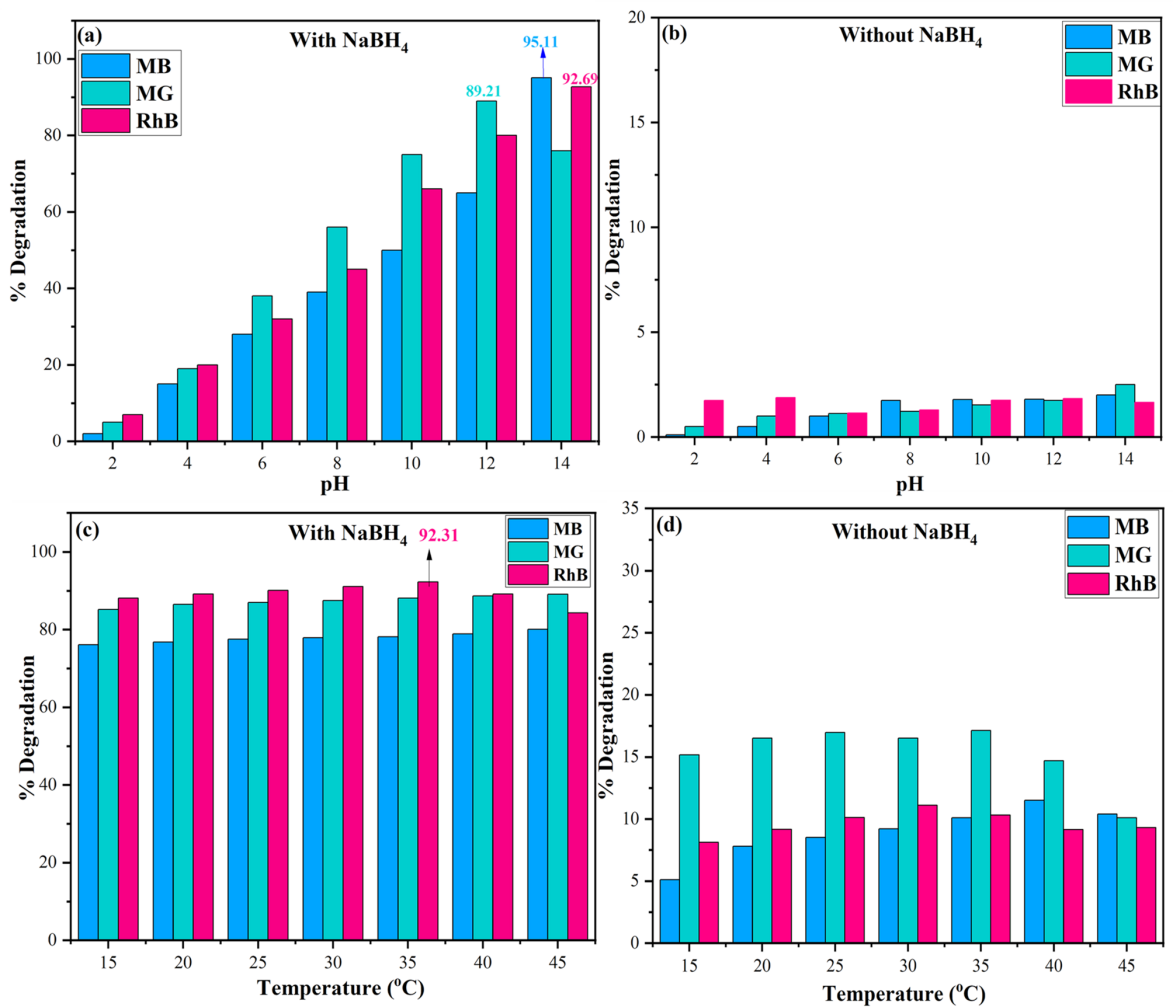
(**a**,**b**) pH factor and (**c**,**d**) Temperature factor of dyes with and without NaBH_4_.

The pH of a solution tends to play an imperative role in the catalytic degradation of countless pollutants. The effect of pH on the catalytic degradation of MB, RhB and MG were studied in between the ranges 2–14 as can be portrayed from Fig. 6a–d. The % degradation of all three of the dyes at pH 3 were 28.12 (MG), 38.71 (MB), and 30.16 % (RhB) and it tends to increase at pH 8 50.12 (MG), 75.18 (MB), and 66.15 % (RhB) then at pH 13 it was 89.1 (MG), 95.12 (MB) and 88.5 % (RhB) respectively. The possibility of enhanced catalytic degradation in the alkaline media is because of the availability of excessive OH^−^ ions in the solution. The removal of MB efficacy is directly proportional to the pH of the solution while keeping other things constant i.e. the concentration of the dye, reducing agent and catalyst^61^. At upper pH values, the exterior of the catalyst become gradually negative because of the existence of OH^−^ ions on the surface therefore ensuring better removal efficacy due to the presence of electrostatic attractive forces with positively charged MB cations^62^. Similar trends were observed in the MG and RhB dyes but less or no degradation was observed when the reducing agent was not introduced to the reaction mixture, similar results were reported by^63^. The catalytic efficiency of the catalyst was examined at seven different temperatures. The experimental lower and higher temperatures were 15 – 45 °C respectively. It was experiential that the catalytic efficiency of the composite degraded the MG, MB and RhB was higher in rates at 45 °C but in the presence of the reducing agent. Therefore, it is safe to say that the nanocatalyst can be used further for the degradation of other dyes and organic pollutants, see Figure (a-d)^64,65^. The results of triplicate readings are mentioned in Table 1.

**Table 1:** Replicate data for the pH and temperature factor for all three dyes with NaBH_4_.

pH	2	4	6	8	10	12	14
MG	5.02	19.08	38.04	56.02	75.11	89.07	76.10
	5.15	19.05	38.06	56.05	75.08	89.10	76.82
	5.07	19.01	38.10	56.11	75.02	89.14	76.50
MB	2.12	15.01	28.11	39.15	50.16	65.18	95.10
	2.05	15.04	28.04	39.05	50.06	65.13	95.16
	2.07	15.07	28.06	39.06	49.96	65.05	95.07
RhB	7.11	20.10	32.01	45.11	66.13	80.19	92.69
	7.14	20.12	32.06	45.08	66.08	80.11	92.51
	7.08	20.15	32.10	45.01	66.01	80.07	92.38
**Temperature**	**15 °C**	**20 °C**	**25 °C**	**30 °C**	**35 °C**	**40 °C**	**45 °C**
MG	85.16	86.51	86.98	87.51	88.12	88.69	89.11
	85.21	86.65	87.12	87.58	88.31	88.72	89.15
	85.14	86.48	86.84	87.42	88.04	88.65	89.04
MB	76.11	76.81	77.51	77.92	78.19	78.91	80.12
	76.15	76.85	77.58	77.87	78.01	78.45	80.02
	76.02	76.61	77.32	77.81	78.25	78.71	80.09
RhB	88.13	89.18	90.12	91.11	92.31	89.15	84.31
	88.04	89.10	90.05	91.02	92.25	89.05	84.11
	88.06	89.14	90.10	91.07	92.11	89.09	84.25

#### Plausible degradation mechanism of dyes

**Fig. 7:**
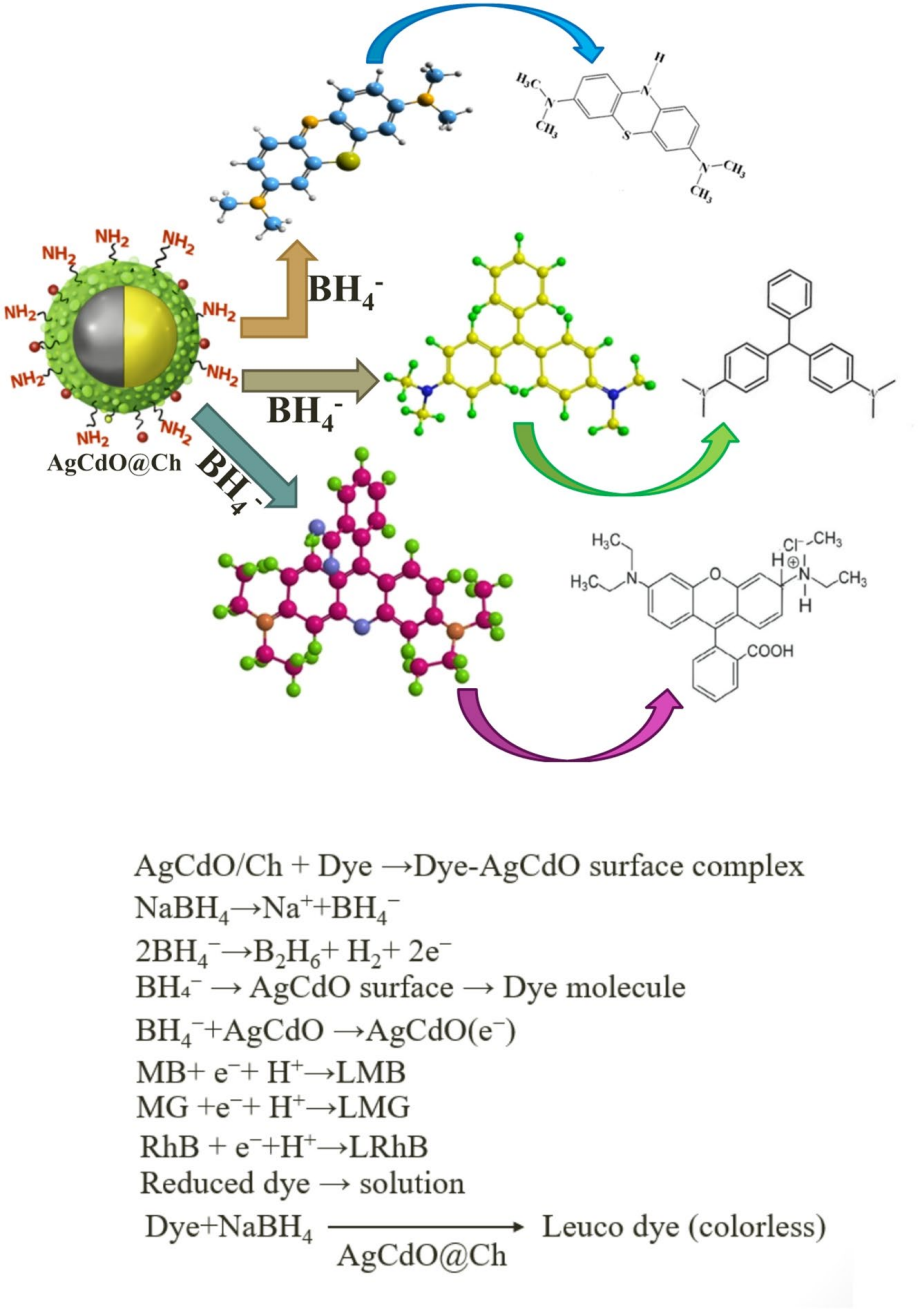
The plausible degradation mechanism of MG, MB and RhB dyes using nanocatalyst and the reducing agent.

The catalytic activity of nanocatalyst using various has been reported herein. AgCdO-BMNPs@Ch displayed reduction of MB, MG and RhB by NaBH_4_. A large active surface area is provided by AgCdO BMNPs while Chitosan contains –OH and –NH_2_ groups which enhances the adsorption of dye molecules via electrostatic forces of attraction, hydrogen bonding, and π–π interactions with the dyes. Both BH_4_^−^ ions and dye molecules adsorbed on the surface of the nanocatalyst. While in aqueous solution, the NaBH_4_ gets dissociated to produce hydride ions and electrons, meanwhile the BH_4_^−^ ions decompose on the surface of the BMNPs producing electrons and hydrogen. These electrons transfer to the surface of the catalyst and make it electron rich. The Ag sites facilitate rapid electron transfer while CdO provide active sites as it is a semiconductor, hence enhancing the charge transfer efficiency. The adsorbed dye molecules receive electrons and hydrogen atoms from the surface of the catalyst and get reduced. The reduced products have weak interaction with the catalyst therefore they desorb into the solution. The catalyst surface become free for the next reaction cycle; similar studies were observed by^66–68^. The scheme of reaction is given below, and Fig. 7 explains the degradation mechanism for all three dyes.

In general, the authors indicated that the leuco form of dyes can be obtained by using NaBH_4_ but the activity does not occur in the absence of catalyst therefore it is a clear indication that both catalyst and NaBH_4_ are vital for the degradation to occur. In the presence of NaBH_4_, AgCdO-BMNPs@Ch acts as a catalyst and reduction of dyes namely MG, MB and RhB to their colorless form occurred. As electron mediators, the catalyst helps in reducing the dyes by transferring electrons from the BH_4_^−^ ions (donor) to dyestuff molecules (electron acceptors). As BH_4_^−^ ions hold to the surface of the catalyst so that the electrons can travel from the donor to the acceptor via BMNPs, therefore the intense color of the dye’s changes to colorless. The intensity of absorption peaks of the dyes significantly drops down even at room temperature confirming the degradation of dyes which can then be monitored by the UV-Vis spectrophotometer^69,70^ (Table 2).

**Table 2:** Comparative study of nanocomposite potential for the reduction of cationic dyes based on time, concentration of the reducing/oxidizing agent, concentration of the dye.

Materials	Method	Dye concentration	Time	Oxidizing/ reducing agent	% degradation	Refs.
AgCd NPs	Catalysis	0.055 mM MG 0.034 mM RhB	4 9	4.1 mM NaBH_4_ 3.5 mM NaBH_4_	96 94	^71^
MgFe_2_O_4_	Catalysis	10 mg/L MG	< 1	10 % H_2_O_2_	100	^72^
AgSr NPs	Catalysis	0.32 mM MG 0.2 mM MO	21 70	22.4 mM NaBH_4_ 20.1 mM NaBH_4_	86.4 96.9	^73^
Cu–Ag NPs	Catalysis	5 mL,50 mg RhB 5 mL,50 mg MO	40 25	5 mg NaBH_4_	95 94	^74^
AgCdO-BMNPs@Ch	Catalysis	1.5 mL (0.02 mM) MB 1.5 mL (0.01 mM) MG 1.5 mL (0.025 mM) RhB	12 16 20	0.5 mL (2 mM) NaBH_4_ 0.5 mL (1 mM) of NaBH_4_ 0.5 mL (1 mM) of NaBH_4_	96.16 92.42 91.62	This study

## Conclusion

Researchers around the globe have reported number of methods for the removal of organic pollutants. In this study, a novel nanocomposite (AgCdO-BMNPs@Ch) was synthesized and characterized employing different techniques such as UV, FTIR, XRD and SEM. The catalytic potential of the composite was studied and it has been confirmed by the experimental results that in the absence of composite, reducing agent was found uanble to degrade the dye. The combination of reducing agent and comopsite resulted in the degradation of dyes (MG, MB and RhB) up to 92.42, 96.16 and 91.62 % respectively. Although, the results were quite promising but one thing we need to consider is that formation of CdO instead Cd as the later one has its own environmental toxicology. Authors have been working on composites containing Cu, Zn and Sr for the removal of same dyes from aqueous medium. These dyes have detrimental effects on human health as well as aquatic life therefore it is the need of the hour to synthesize materials which are stable, safe and can with-stand various acidic and basic conditions; also, their reusability is the key factor in addressing these persistent issues. Therefore, it is safe to say that the reported composite can further be used for environmental remediation. It is recommended to investigate the potential of the composite against anionic dyes or organic pollutants like 4-nitrophenol, and Cr(VI) etc.

## Data Availability

Data will be made available on a reasonable request.
